# The Spinal Cord Injury Program in Exercise (SCIPE) study: study protocol for a randomized controlled trial evaluating teleexercise programs for people with spinal cord injury

**DOI:** 10.1186/s13063-021-05474-4

**Published:** 2021-08-19

**Authors:** Hui-Ju Young, Tapan Mehta, Yumi Kim, Sangeetha Padalabalanarayanan, Chia-Ying Chiu, James H. Rimmer, Mohanraj Thirumalai

**Affiliations:** 1 UAB/Lakeshore Research Collaborative, School of Health Professions, University of Alabama at Birmingham, Birmingham, AL USA; 2 Department of Health Services Administration, School of Health Professions, University of Alabama at Birmingham, Birmingham, AL USA; 3 Department of Physical Therapy, School of Health Professions, University of Alabama at Birmingham, Birmingham, AL USA

**Keywords:** Spinal cord injury, Physical activity, Disability, Teleexercise, Quality of life

## Abstract

**Background:**

Many people with spinal cord injury (SCI) have limited access to tailored, readily available exercise resources. As a result, exercise remains an underutilized treatment strategy for improving health and function in people with SCI. The purpose of this study is to test the effectiveness of two remotely delivered exercise programs for people with SCI.

**Methods:**

The Spinal Cord Injury Program in Exercise (SCIPE) study is a three-arm adaptive randomized controlled trial examining two 8-week teleexercise interventions: Movement-to-Music (M2M) and Standard Exercise Training (SET), compared to Attention Control (AC) in 327 adults with SCI. The primary outcome is change in physical activity level at post 8-week intervention. The study contains two interim analyses. The first interim analysis will assess feasibility metrics of the protocol after 36 participants complete the 8-week intervention period. The second interim analysis will examine two effectiveness comparisons: SET vs. AC and M2M vs AC, after 165 participants complete the intervention period. Early termination of the intervention arm(s) will take place when non-significant findings are found in the corresponding intervention(s). Incorporation of such interim analysis enhances trial efficiency by dropping the intervention(s) that deemed ineffective. It provides ethical benefits and allows allocation of additional resources to explore the effective intervention(s).

**Discussion:**

Delivery of teleexercise programs may be an effective strategy for addressing transportation barrier to exercise resources and increasing physical activity level and quality of life in people with SCI.

**Trial registration:**

ClinicalTrials.gov Identifier NCT03925077. Registered trial name: Spinal Cord Injury Program in Exercise (SCIPE). Registered on April 23rd, 2019.

## Administrative information

The order of the items has been modified to group similar items (see http://www.equator-network.org/reporting-guidelines/spirit-2013-statement-defining-standard-protocol-items-for-clinical-trials/).

**Table:** 

Title {1}	The Spinal Cord Injury Program in Exercise (SCIPE) Study: Study Protocol for a Randomized Controlled Trial Evaluating Teleexercise Programs for People with Spinal Cord Injury.
Trial registration {2a and 2b}.	ClinicalTrials.gov Identifier: NCT03925077. Registered on April 23^rd^, 2019.
Protocol version {3}	Protocol Version 1; September 2020.
Funding {4}	National Institute on Disability, Independent Living, and Rehabilitation Research (NIDILRR). Grant number 90REGE0002.
Author details {5a}	^1^"UAB/Lakeshore Research Collaborative, School of Health Professions, University of Alabama at Birmingham, Birmingham, AL ^2^Department of Health Services Administration, School of Health Professions, University of Alabama at Birmingham, Birmingham, AL ^3^Department of Physical Therapy, School of Health Professions, University of Alabama at Birmingham, Birmingham, AL
Name and contact information for the trial sponsor {5b}	RERC RecTech University of Alabama at Birmingham, SHPB 331, 1720 2nd Ave S Birmingham, AL 35294-3361
Role of sponsor {5c}	The sponsor plays no part in study design, data collection, data management, data analysis, data interpretation, and decision to submit results for publication.

## Introduction

### Background and rationale {6a}

Many people with spinal cord injury (SCI) live in rural communities and other geographically isolated areas where access to fitness facilities and outdoor recreation venues involve long commutes or costly transportation [1], which is one of the most common exercise barriers reported by people with physical disabilities [2]. As a result, exercise remains an underutilized intervention for improving health and function in people with SCI despite its proven effects to reduce pain, fatigue, falls risk, and other secondary health conditions [3–5]. Latimer and coworkers [6] reported that people with SCI spent less than 2% of their waking hours engaged in any type of structured exercise or leisure time physical activity, and concluded that physical inactivity is a serious public health issue in this population. The inactivity can lead to further physical deconditioning and eventually result in a cycle of reduced mobility and increased secondary health conditions [7]. There is a need for implementing effective program delivery strategies that can promote sustainable exercise behavior in people with SCI.

Delivery of home-based exercise, or what we refer to as teleexercise, for people with SCI to participate in the comfort of their home can potentially promote engagement in physical activity by addressing transportation barriers as well as other barriers including inaccessible fitness facilities and expensive gym memberships [1, 8, 9]. Dallolio et al. [10] examined functional and clinical outcomes in participants with SCI who received 6-month telerehabilitation intervention and those who received standard care. They found that participants in the telerehabilitation group had higher satisfaction with care compared to the standard care group. Thus, teleexercise is a promising area of technology that could encourage sustainable exercise participation in people with SCI [11–13].

Today, with rapid advances in telehealth technology [14], it is now possible to deliver customized teleexercise programs on a large scale. A survey published in 2008 indicated that approximately 69.2% of people with SCI used a computer at home and other locations including school, work, library, or internet café, with 61.3% seeking information on health and disability online [15]. This merger of health care and technology is one of the most exciting and rapidly growing areas in exercise and rehabilitation and has substantial implications for conducting high fidelity exercise trials for people with SCI.

Thus, the purpose of the Spinal Cord Injury Program in Exercise (SCIPE) study is to examine two 8-week teleexercise interventions: Movement-to-Music (*M2M*) and Standard Exercise Training (*SET*), with 327 adults with SCI.

### Objectives {7}

The SCIPE study aims to:

1. Examine change in physical activity levels after the 8-week *M2M* and *SET* interventions. We hypothesize that participants in *M2M* and *SET* will have a significant increase in physical activity compared to an Attention Control (*AC*) group post-intervention.
2. Examine the effects of the *M2M* and *SET* interventions on health and quality of life outcomes. We hypothesize that participants in *M2M* and *SET* will have significant increases in sleep quality and quality of life and decreases in pain and fatigue compared to *AC* post-intervention. Exercise enjoyment in *M2M* and *SET* participants will also be explored.
3. Evaluate the demographic (age, race, sex), clinical (level of injury, type of injury), and psychosocial (social support, outcome expectations, self-efficacy, self-regulation) variables of two participant groups: (1) compliant participants who completed ≥ 50% of the intervention, and (2) noncompliant participants who completed post-testing but < 50% of the intervention or who did not complete post-testing.

### Trial design {8}

**Fig. 1:**
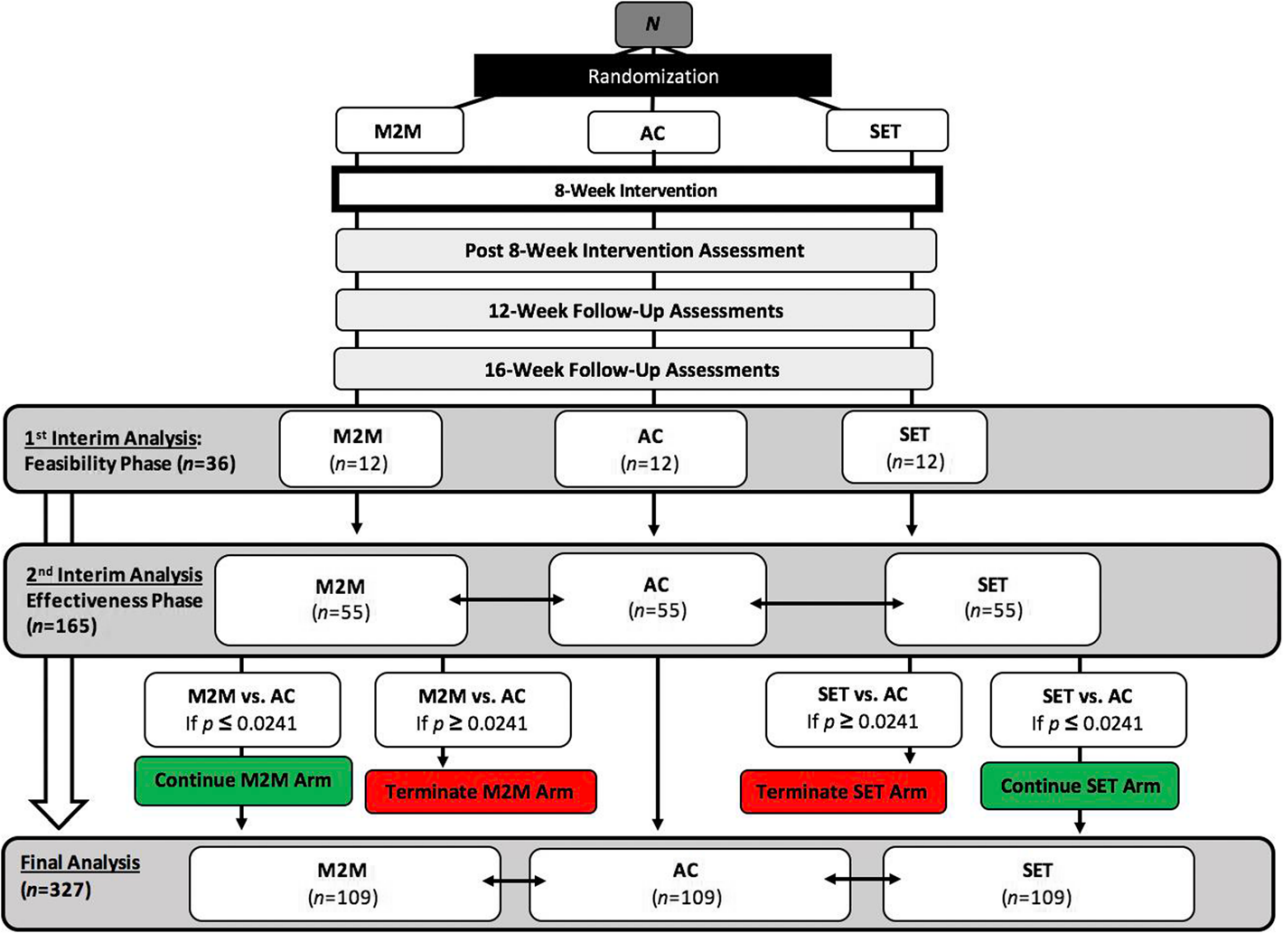
Overall structure of the SCIPE study

The SCIPE study is designed as a three-arm randomized controlled trial that utilizes a sequential design and contains two interim analyses. Eligible and enrolled participants will be randomized into one of three study arms: *M2M*, *SET*, and *AC*. The two interim analyses are mapped with two phases of the study, which are the feasibility phase and the effectiveness phase. The feasibility phase will include the first 36 enrolled participants, with the first interim analysis assessing feasibility metrics of the protocol after the 36 participants complete the 8-week intervention period and post-intervention assessment. The effectiveness phase will include 165 participants, with the second interim analysis examining two effectiveness comparisons: *SET* vs. *AC* and *M2M* vs. *AC*, after the 165 participants complete the intervention period and post-intervention assessment. When an intervention is found to be statistically ineffective, the corresponding intervention arm will be terminated. Early termination of an ineffective intervention promotes trial efficiency by optimizing the utilization of resources in the effectiveness intervention. It also allows a higher probability of participants receiving an intervention that is effective. The overall study structure is illustrated in Fig. 1.

## Methods: Participants, interventions, and outcomes

### Study setting {9}

The study will take place entirely online, including collection of all outcome measures and delivery of the interventions. All outcome measures will be collected using electronic surveys through the Research Electronic Data Capture (REDCap). The M2M and SET interventions will be delivered through a secure teleexercise platform, the SCIPE website, that is specifically designed and built for the study.

### Eligibility criteria {10}

Individuals are eligible to participate in the study after meeting all inclusion criteria, which include (1) diagnosed with a SCI resulting in incomplete or complete (C5 and below) paraplegia and tetraplegia, (2) between ages of 18 and 65 years, (3) demonstrate readiness to physical activity by completing the Physical Activity Readiness Questionnaire for Everyone (PAR-Q+) [16], (4) obtain medical clearance if required by PAR-Q+, and (5) converse in and read English. Exclusion criteria include (1) no broadband internet access, (2) significant visual acuity that prevents seeing a computer screen to follow home exercise program, and (3) currently pregnant.

### Who will take informed consent? {26a}

Upon completion of the screening process, eligible individuals will receive an electronic consent form via REDCap and will officially enroll in the study after providing informed consent.

### Additional consent provisions for collection and use of participant data and biological specimens {26b}

The consent form asks if participants are willing to be contacted for future research opportunities. Participants can say no to this and still participate in the SCIPE study.

## Interventions

Enrolled participants who complete baseline assessment will be randomly assigned to *M2M*, *SET*, or *AC*. After randomization, each participant will receive an invitation email with a link to the SCIPE website and will be instructed to complete a series of steps before being given access to the website content. The steps include (1) creating a password, (2) completing a user profile. (3) answering questions on physical function and presence of joint pain, and (4) setting up an exercise schedule and calendar reminder. The website content provided to participants is based on their assigned study arm. For participants who are in the *M2M* and *SET* arms, their responses to the physical function and joint pain questions will be used to tailor the exercises they will receive in their assigned intervention. The questions are presented in Table 1.

**Table 1:** Physical function and joint pain questions

Item #	Question	Response options
1.	Please select one that best describes your functional level.	I can use both arms and legs to exercise.
		I can use both arms to exercise with good trunk control.
		I can use both arms to exercise with little or no trunk control.
2.	Do you have any pain in your lower back that would limit you from doing exercise in that area?	Yes
		No
3.	Do you have any pain in your shoulder(s) that would limit you from doing exercise in that area?	Yes
		No
4.	Do you have any pain in your knee(s) that would limit you from doing exercise in that area?	Yes
		No

### Explanation for the choice of comparators {6b}

The *M2M* and *SET* interventions are designed to gradually build participants’ physical activity level toward the US-recommended physical activity guidelines for people with SCI [17, 18] and the general adult population [19]. Both interventions are structured with the same exercise dosage and consist of exercises that target four fitness components: range of motion, muscular strength, cardiorespiratory endurance, and balance. Table 2 highlights the general structure of the interventions. The difference between *M2M* and *SET* is that the exercises in *M2M* are choreographed into movement sequences that are performed with music.

**Table 2:** General intervention structure

Intervention structure	Session component workout time (in minutes)							
Week	1	2	3	4	5	6	7	8
**Session component**								
**Upper body range of motion**	5	5	5	5	5	5	5	5
**Lower body range of motion**	5	5	5	5	5	5	5	5
**Muscular strength**		5	5	5	5	5	5	5
**Aerobic**			5	5	10	15	20	20
**Functional strength**				5	5	5	5	5
**Cool down/breathing**			5	5	5	5	5	5
**Total**	**10**	**15**	**25**	**30**	**35**	**40**	**45**	**45**

### Intervention description {11a}

The *M2M* and *SET* interventions include three sessions per week for a total of 8 weeks and are delivered through pre-recorded exercise videos that progress from 10-min exercise time per session in week 1 to 45 min per session in weeks 7 and 8. Prior to beginning the intervention, participants will receive a set of wrist weights from the study staff. Participants will be required to watch an introductory video, where basic movement and posture, equipment use, and exercise safety are explained. Each session begins with a seated range of motion exercises, followed by muscular strengthening exercises performed using the wrist weights, cardiorespiratory endurance, and balance exercises performed either seated or standing with or without the support of a chair. The session ends with cool-down exercises that emphasize breathing. For participants who indicate being able to use both arms and legs to exercise with no knee pain via the physical function and join pain questions (Table 1), they will receive cardiorespiratory endurance and balance videos demonstrating the exercises in a standing position. For participants who indicate being able to use both arms and legs to exercise but having knee pain or being able to use both arms to exercise with or without trunk control, they will receive the cardiorespiratory endurance and balance videos demonstrating in a seated position. For participants who indicate having shoulder and/or back pain, a safety clip reminding them about proper exercise posture for their shoulder joints and back will be given at the beginning of each session. Equipment used in both interventions include a chair and wrist weights. In addition, participants in all three arms will receive educational articles through the SCIPE website. A new article will be uploaded every week for 8 weeks. The article topics include physical activity recommendations, injury prevention, benefits of exercise, exercise goal settings and self-monitoring, and maintenance of an active lifestyle.

#### Movement-to-Music (*M2M*)

The *M2M* intervention is based on positive effects of exercise and music on both physiological and psychosocial outcomes in people with disabilities [20–23]. The intervention uses combinations of movement forms choreographed to music to create movement routines. Every routine is specifically designed to target one of the four fitness components with a set range of movement tempo.

#### Standard Exercise Training (*SET*)

The *SET* intervention is based on the National Center on Health, Physical Activity, and Disability (NCHPAD) 14-Weeks to a Healthier You program launched in 2008. It involves standard exercises that are performed in both standing and seated positions.

#### Attention Control (*AC*)

Participants in the *AC* group will not have access to any exercise videos. They will have access to the weekly educational articles on the SCIPE website.

### Criteria for discontinuing or modifying allocated interventions {11b}

The allocated interventions will not be modified if requested by participants. Participants may choose to discontinue the intervention or withdraw from the study for any given reason, including no longer being interested in the study or being assigned to a study arm that is not a preference.

### Strategies to improve adherence to interventions {11c}

The SCIPE study incorporates several strategies to improve participant adherence to the intervention and study. First, after participants indicate their exercise time preference when they first enter the SCIPE website, a calendar invite will be created for participants to incorporate the scheduled exercise sessions into their personal calendar. Second, all participants will receive weekly notifications when new website content, including exercise videos and articles for *M2M* and *SET* participants and articles for *AC* participants, are uploaded to the SCIPE website. The weekly notifications, depending on participants’ preferences, can be delivered through either text message or email [24, 25]. Third, during the intervention period, exercise adherence will be monitored through the frequency of a website login and video watching duration. Study staff will contact participants who enter the website for less than one time a week in the past 2 weeks. Fourth, to encourage participation of the weekly assigned exercise videos and articles, participants will receive electronic badges when they complete the weekly assignments. Fifth, through the SCIPE website, participants will be able to add each other as friends and communicate about their exercise experience and progress. Sixth, to ensure any questions or technical issues can be addressed in a timely manner, participants will be able to reach the study team via Contact Staff function on the SCIPE website. When participants utilize the Contact Staff function, the study team will be notified immediately via email and will contact the participants within 2 business days.

### Relevant concomitant care permitted or prohibited during the trial {11d}

Not applicable, no concomitant care or interventions are prohibited during the trial.

### Provisions for post-trial care {30}

No compensation is offered to those who might suffer from harm or injury from study participation. Participants will continue having access to the exercise videos and/or articles on the SCIPE website after completion of their study participation.

### Outcomes {12}

All participants will be asked to complete a baseline assessment, a post 8-week intervention assessment, a 12-week follow-up assessment, and a 16-week follow-up assessment. Each assessment includes a list of questionnaires that will be delivered to participants as an electronic survey packet via REDCap. The questionnaires and measures that are used to assess participant demographics and health history as well as primary and secondary outcomes are described in Table 3. The primary outcome is change in physical activity after the 8-week intervention. The secondary outcomes at 8 weeks include changes in pain intensity, pain interference, sleep quality, fatigue, health-related quality of life, and exercise enjoyment. The mediators include exercise adherence and four social cognitive theory constructs: self-efficacy, self-regulation, social support, and outcome expectations.

**Table 3:** Outcome measures of the SCIPE study

Role	Outcome measures	Instrument	Collection time points
**Primary outcome**	Physical activity	Leisure Time Physical Activity Questionnaire for People with Spinal Cord Injury [20]	Baseline, 8-week, 12-week, and 16-week
**Secondary outcome**	Pain intensity	NIH PROMIS [21] Pain Intensity Adult Short Form 3a	Baseline, 8-week, 12-week, and 16-week
	Pain interference	NIH PROMIS Pain Interference Adult Short Form 8a [22]	
	Sleep quality	NIH PROMIS Sleep Disturbance Adult Short Form 8a [23]	
	Fatigue	NIH PROMIS Fatigue Adult Short Form 7a [24]	
	Health-related quality of life	NIH PROMIS 10 Global Health Items [25] NIH PROMIS Ability to Participate in Social Roles and Activities Short Form 8a	
	Exercise enjoyment	Physical Activity Enjoyment Scale [26]	
**Mediators**	Exercise adherence	Percentage of # of attended exercise sessions	8-week intervention period
	Exercise self-efficacy	Exercise Self-efficacy Scale [27]	Baseline, 8-week, 12-week, and 16-week
	Exercise self-regulation	Exercise Goal-setting Scale [28]	
	Social support for exercise	Social Provisions Scale [29]	
	Outcome expectations for exercise	Multidimensional Outcome Expectations for Exercise Scale [30]	

In addition, up to 50 participants will be randomly selected to participate in a follow-up interview after the 8-week intervention to assess their experiences using the SCIPE website and participating in the exercise interventions.

### Participant timeline {13}

Table 4 shows the schedule of enrollment, randomization, interventions, and assessments for the SCIPE study participants.

**Table 4:**
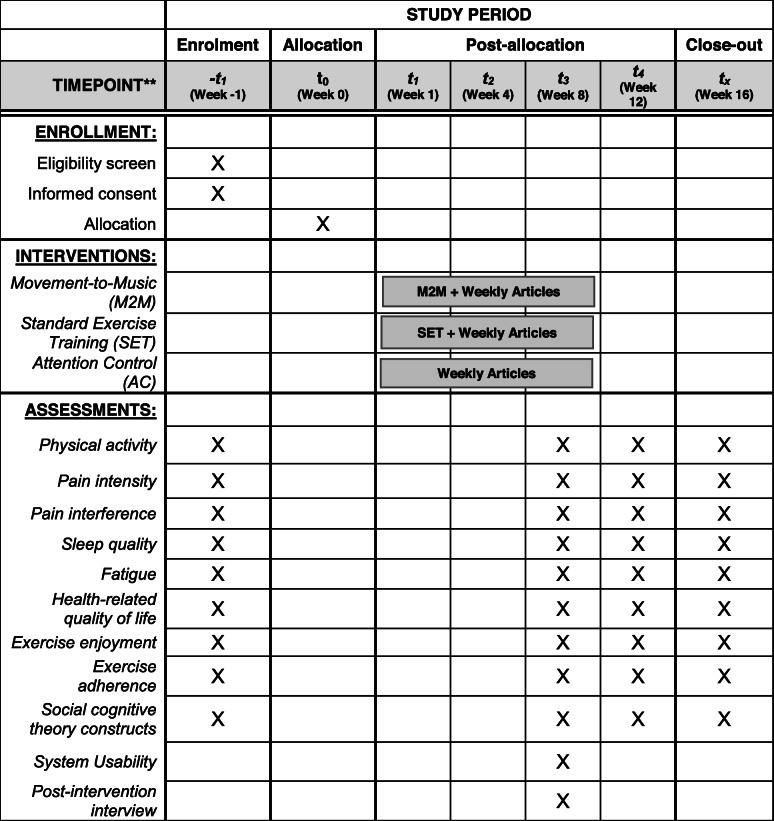
Schedule of enrollment, randomization, interventions, and assessments for the SCIPE study participants

### Sample size {14}

Our intent, therefore, is to recruit a total of 327 participants, 109 in each of the groups (*M2M*, *SET*, *AC*). An a priori power calculation was performed for the primary outcome (physical activity) using PASS 15. We assumed an ANCOVA with a modest correlation of 0.6 between baseline covariate and outcome based on our estimates from a previous study. To account for the second interim look-up with rules to adapt the design of the trial, the type 1 rate assumed was 0.033. To achieve an 80% power for a moderate effect size of 0.4 (Cohen’s *d*) and with aforementioned assumptions yielded a total sample size of 216 completers. The chosen effect size is based on previously published papers [26, 27]. Assuming a 34% attrition rate, we will recruit and randomize 327 participants. Our choice of attrition rate is based on a 26.1% attrition found in a home-based exercise intervention [28] with the intent to be conservative in our assumption of attrition.

### Recruitment {15}

Potential participants will be recruited nationwide by disseminating recruitment information through the study website (https://scipe.org). Study information will also be distributed through an electronic newsletter of a large 501C exercise facility for people with SCI and other disabilities (Lakeshore Foundation, www.lakeshore.org), social media channels, and through the National Center on Health, Physical Activity and Disability, a Center funded by the US Centers for Disease Control and Prevention (www.nchpad.org). If potential participants are interested in participating, they will be provided with a link that will direct them to the study website where they can find and complete a pre-study screening form.

## Assignment of interventions: Allocation

### Sequence generation {16a}

The randomization sequence is generated by a study biostatistician (TM) who is not involved in intervention development a priori using a computer-generated random schedule (SAS version 9.4) with 1:1:1 allocation ratio.

### Concealment mechanism {16b}

The randomization sequence is uploaded to a randomization module in REDCap.

### Implementation {16c}

Randomization will be performed entirely in REDCap by study staff after a participant completes baseline assessment.

## Assignment of interventions: Blinding

### Who will be blinded {17a}

Due to the nature of the intervention, it is not plausible to blind participants and study staff who enroll participants and monitor the intervention delivery. However, all data analyses and reporting will be performed by study investigators and staff who are blinded to study arm assignments.

### Procedure for unblinding if needed {17b}

Study personnel who are responsible for analyzing data and reporting results will remain blinded throughout the study period.

## Data collection and management

### Plans for assessment and collection of outcomes {18a}

All data will be collected directly through electronic questionnaire packets delivered via REDCap at baseline, post-intervention, 12-week follow-up, and 16-week follow-up assessments. All question items of the questionnaire packets are made as required to answer to prevent data missingness. Each questionnaire packet is also set to be delivered to participants’ email inbox three times, separated by 7 days, if it is not completed. A follow-up phone call will be made if participants do not complete the packet after 14 days to ensure participants receive the packet and remind them to complete it within the next 7 days.

### Plans to promote participant retention and complete follow-up {18b}

Financial incentives will be provided to participants for completing study assessments, which include $25 for baseline, $30 for 8-week post-intervention, and $20 each for 12- and 16-week follow-ups. In addition, participants who participate in the follow-up interview will receive an additional $15 incentive.

### Data management {19}

All data will be collected electronically. The University Information Technology Research Computing servers will be used as a central location for data processing and management of electronic data. Electronic data will be stored in REDCap. REDCap is a software program that was developed by Vanderbilt University, with collaboration from a consortium of institutional partners (including UAB) and the NIH National Center for Research Resources, for electronic collection and management of research and clinical trial data. REDCap data collection projects rely on a thorough study-specific data dictionary defined in an iterative self-documenting process by all members of the study team. As part of the data dictionary development process, individual fields can be denoted as “identifiers.” When exporting a de-identified dataset, these variables are omitted. Additionally, the data export tool also allows for the shifting of dates for a limited data set export.

### Confidentiality {27}

REDCap is 21 CRF Part 11 capable. Currently, REDCap installations support electronic signatures by positively identifying the user through a unique username and password combination. The study team will enter all source document data into REDCap under the supervision of the biostatistician (TM). Access to the REDCap database will be given to the study personnel only, including the biostatistician and data entry/management personnel. Data being analyzed will be exported in de-identified format. Identities of participants will not be revealed in the presentation or publication of any result from this project. Assistants and others working on this project will be educated about the importance of strictly protecting subjects’ rights to confidentiality. Participants will be informed of law-mandated instances in which confidentially could be breached.

### Plans for collection, laboratory evaluation, and storage of biological specimens for genetic or molecular analysis in this trial/future use {33}

Not applicable, no biological specimens will be collected in the study.

## Statistical methods

### Statistical methods for primary and secondary outcomes {20a}

For the *primary and secondary aims*, we will conduct a series of repeated ANOVA tests to examine changes in physical activity and self-reported health after the 8-week intervention. A Fisher’s LSD will be conducted to compare means between groups further when appropriate and the corresponding effect sizes (Cohen’s *d*) will be calculated for each outcome. A Student’s t-test will be used to compare exercise enjoyment (week 8) between *M2M* and *SET* groups. Exercise adherence will be analyzed using descriptive statistics. For the *tertiary aim*, multiple logistic regression will be conducted to examine the relationships between the independent variables (age, race, sex, level of injury, type of injury, social support, outcome expectations, self-efficacy, and self-regulation), and dependent variables (compliance and non-compliance). Statistical tests will be performed at an overall alpha level (family-wise error rate) of 0.05.

### Interim analyses {21b}

The first interim analysis will evaluate feasibility metrics of the protocol. The feasibility metrics include participant satisfaction and usefulness of using the SCIPE website, which will be assessed through two questionnaires: the System Usability Scale [29] and the Health IT Usability Evaluation Scale [30, 31]. Semi-structured interviews will also be conducted and will include predominantly open-ended questions to allow participants to express their experiences and opinions toward the SCIPE website. All interviews will be audiorecorded and will then be transcribed by a professional transcription service, Rev.com. Results from this first interim analysis will be used to refine the protocol. The second interim analysis will evaluate the effectiveness of the interventions by comparing *M2M* with *AC* and *SET* with *AC* after 165 participants are randomized across the three arms. If one of these two comparisons does not yield statistically significant findings at an alpha level of 0.0241, the corresponding intervention arm will be terminated. To account for the inferential analyses, the final analysis will be conducted at a significance level of 0.033.

### Methods for additional analyses (e.g., subgroup analyses) {20b}

Not applicable, no additional subgroup analyses are planned.

### Methods in analysis to handle protocol non-adherence, and any statistical methods to handle missing data {20c}

Missing data will be imputed using multiple imputation where necessary, with assumption that the missingness mechanism is missing at random.

### Plans to give access to the full protocol, participant level-data, and statistical code {31c}

This paper provides the full protocol. Readers should contact the authors if interested in other data or documentation of the study.

## Oversight and monitoring

### Composition of the coordinating center and trial steering committee {5d}

Not applicable, the study does not include a coordinating center and trail steering committee.

### Composition of the data monitoring committee, its role and reporting structure {21a}

The study does not have a Data Monitoring Committee. The study biostatistician (TM) will be responsible for overseeing data entry/data management, study design fidelity, data sharing, and preparation of peer-reviewed manuscripts (data analysis). The study investigator (H-JY) will be responsible for monitoring the data collection process throughout the study process.

### Adverse event reporting and harms {22}

The SCIPE study will monitor adverse events (AEs) and report them based on four types defined by the Behavior Change Consortium of the National Institutes of Health [32]. The four types of AEs are (1) falls, (2) cardiovascular-related episodes, (3) musculoskeletal-related events, and (4) health care use. All AEs will be assessed for severity and causality and will be reported to IRB and relevant regulatory bodies when necessary.

### Frequency and plans for auditing trial conduct {23}

Not applicable, the study does not contain plans for auditing trial conduct.

### Plans for communicating important protocol amendments to relevant parties (e.g., trial participants, ethical committees) {25}

When important protocol amendments are necessary, the study team will notify and communicate with the program officer of the funding agency. Approvals from the program officer will be obtained prior to amending the protocols.

### Dissemination plans {31a}

Findings from this study will be shared publicly and disseminated mainly by publication in peer-reviewed journals and conference presentations. Findings will also be disseminated through NCHPAD and Lakeshore Foundation social media outlets.

## Discussion

People with SCI need convenient access to readily available exercise programs that can help them achieve recommended physical activity level. The transportation barrier experienced by many people with SCI provides strong justification for the development of a comprehensive technology platform for providing enjoyable, home-based exercise tailored to the needs of people with SCI. From the perspective of people with SCI, there are many potential benefits of delivering teleexercise programs. Most often noted is their convenience, allowing participants to exercise anywhere and anytime that works best for them. It also eliminates travel time, which can be several hours to and from a fitness facility when using specialized transportation services.

To this end, the SCIPE study will test strategies that can help address several barriers to exercise participation among people with SCI. First, there is a lack of access to exercise and fitness facilities for many people with SCI living in geographically diverse regions across the United States. While telehealth is increasingly becoming an integral part of healthcare [14], we are not aware of any scalable technology platform that addresses the unique needs of people with SCI in prescribing tailored, home-based exercise. Second, when healthcare providers identify health issues in patients with SCI, it is unclear what, if any, options they have for recommending exercise programs to their patients. With transportation and program costs being two of the most common barriers to exercise reported among people with SCI [4, 33, 34], teleexercise programs hold strong potential for reaching people with SCI and promoting sustainable exercise behavior. Third, SCI has one of the highest rates of sedentary behavior compared to other disability groups because of limited standing and walking activity [4, 5, 34]. People with SCI can achieve much higher levels of health and quality of life if provided with tailored exercise resources over a supportive technology platform. The ultimate goal of the SCIPE study is to find effective strategies to help guide and shape the behavior of people with SCI towards higher levels of regular and sustainable exercise participation.

## Trial status

Protocol Version 1, September 2020. Recruitment started in February 2021. The trial is expected to be completed in September 2022.

## Data Availability

All data that will be used or analyzed in the study will be supplied upon reasonable request.

## Abbreviations

SCIPE: Spinal Cord Injury Program in Exercise
SCI: Spinal cord injury
M2M: Movement-to-Music
SET: Standard Exercise Training
NCHPAD: National Center on Health, Physical Activity, and Disability
AC: Attention Control
PROMIS: Patient-Reported Outcomes Measurement Information System
LTPAQ-SCI: Leisure Time Physical Activity Questionnaire for People with Spinal Cord Injury
REDCap: Research Electronic Data Capture
ANCOVA: Analysis of Covariance
